# Atypical speech versus non-speech detection and discrimination in 4- to 6- yr old children with autism spectrum disorder: An ERP study

**DOI:** 10.1371/journal.pone.0181354

**Published:** 2017-07-24

**Authors:** Alena Galilee, Chrysi Stefanidou, Joseph P. McCleery

**Affiliations:** 1 School of Psychology and Neuroscience, Dalhousie University, Halifax, Nova Scotia, Canada; 2 School of Psychology, University of Birmingham, Edgbaston, Birmingham, West Midlands, United Kingdom; 3 Center for Autism Research, Children’s Hospital of Philadelphia, Philadelphia, Pennsylvania, United States of America; Vanderbilt University, UNITED STATES

## Abstract

Previous event-related potential (ERP) research utilizing oddball stimulus paradigms suggests diminished processing of speech versus non-speech sounds in children with an Autism Spectrum Disorder (ASD). However, brain mechanisms underlying these speech processing abnormalities, and to what extent they are related to poor language abilities in this population remain unknown. In the current study, we utilized a novel paired repetition paradigm in order to investigate ERP responses associated with the detection and discrimination of speech and non-speech sounds in 4- to 6—year old children with ASD, compared with gender and verbal age matched controls. ERPs were recorded while children passively listened to pairs of stimuli that were either both speech sounds, both non-speech sounds, speech followed by non-speech, or non-speech followed by speech. Control participants exhibited N330 match/mismatch responses measured from temporal electrodes, reflecting speech versus non-speech detection, bilaterally, whereas children with ASD exhibited this effect only over temporal electrodes in the left hemisphere. Furthermore, while the control groups exhibited match/mismatch effects at approximately 600 ms (central N600, temporal P600) when a non-speech sound was followed by a speech sound, these effects were absent in the ASD group. These findings suggest that children with ASD fail to activate right hemisphere mechanisms, likely associated with social or emotional aspects of speech detection, when distinguishing non-speech from speech stimuli. Together, these results demonstrate the presence of atypical speech versus non-speech processing in children with ASD when compared with typically developing children matched on verbal age.

## Introduction

Autistic spectrum disorder (ASD) is a neurodevelopmental disorder characterised by impairments in social interaction, communication, and restricted or repetitive interests and behaviours [1]. ASD is a heterogeneous disorder—individuals’ degree of impairment varies widely in the core areas of language, cognition, and social-cognitive functioning [2, 3]. Consistent features of ASD include poor social orienting and joint attention skills [4,5]. In addition, previous behavioural research has shown that, unlike typically developing (TD) children, children with ASD do not demonstrate a reliable preference for their mother’s voice [6]. The results of another study extended this finding and reported a more general lack of preference for human voices in children with ASD [7]. In addition to evidence revealing reduced behavioral orienting to human voices, Kuhl and colleagues further found that variability in social orienting in the autism group in their study was related to the children’s speech sound (phonetic) discrimination skills [8]. Along with other findings, these results have been interpreted as support for the hypothesis that failure to attend to social stimuli is an important aspect of early development in autism, causally contributing to deficits in both social interaction and language skills [4,5, 9, 10]

Čeponiene and colleagues utilised the MMN paradigm in order to examine the neural mechanisms of attentional orienting to both speech and non-speech stimulus changes in school-age (6- to 12- years old) children with autism [11]. The results showed that while the control group exhibited the attentional orienting responses to rarely presented, frequency contrast stimuli in all conditions that the authors predicted at approximately 300 ms (P3a component activity), children with autism failed to exhibit these (P3a, attentional orienting) responses during the speech contrast conditions [11]. These findings further suggest that impairments in the neural systems that mediate involuntary orienting to changes in sounds may be specific to the processing of speech stimuli in children with ASD.

In a follow-up to Čeponiene and colleagues’ study, Whitehouse and Bishop [12] utilised a different variation of the MMN paradigm in 7- to 14- year old children, in which they presented rare novel speech stimuli within a stream of repetitive non-speech stimuli and, separately, rare novel non-speech stimuli in the context of a repetitive stream of speech stimuli. They found that P3a responses were larger in children with autism relative to controls in the repetitive non-speech condition (rare speech sound), whereas their P3a responses were smaller relative to controls in the repetitive speech condition (rare non-speech sound). These group differences, however, were not observed during an active condition in which children were required to pay attention to the sounds in order to perform a behavioural task. In addition, the results of a more recent study utilizing the MMN paradigm demonstrated that processing of emotional components of speech, such as prosody, is diminished at different levels of processing in children with ASD. More specifically, between-group differences in the MMN and P3a component activity were observed in response to a deviant emotional speech stimulus [13]. Taken together, these results suggest that attentional orienting to, and detection of, speech sounds is not universally impaired in children with ASD. Instead, these results suggest that these children may exhibit deficient involuntary orientation to, and diminished processing of repetitive and emotional speech [12–15].

The results of other studies suggest that autism may also be characterized by atypical lateralization of processing for both speech and non-speech sounds. For example, it has been proposed that right-lateralized networks involved in pre-attentive arousal auditory processes are compromised in school-aged children with ASD [16]. This is consistent with the results of an MEG study in which reduction in right-lateralized auditory cortex responses to non-speech sounds, reflected in the amplitudes of P100m auditory component, was observed in school-aged children with ASD relative to controls [17]. Additionally, Orekhova and colleagues found that smaller amplitude P100 activity over the right hemisphere was associated with the severity of sensory dysfunction in this population. Atypical right hemisphere responses were also uncovered when children with ASD were presented with more complex linguistic stimuli [18]**.** Specifically, Brennan and colleagues found that school-aged children with ASD exhibited enhanced sensitivity to processing violations in English phonemes, reflected in right hemisphere activation, which was absent in controls. In line with this finding, speech perception in younger children with ASD was characterised by an atypically increased right hemisphere activation during the perception of speech [19]. Taken together, these previous findings suggest atypical right hemisphere responses to both speech (enhanced processing) and non-speech sounds (reduced processing) in children with ASD, relative to controls.

Altogether, the evidence collected to date suggests a diminished response to speech stimuli compared with non-speech sounds, as well as atypical lateralization of neural activity to both speech and non-speech stimuli, in children with ASD. However, brain mechanisms underlying neural atypicalities in speech processing, and particularly speech versus non-speech detection, in children with ASD remain largely unknown. It is difficult to draw a big picture of speech processing dysfunctions in ASD when combining previous findings, since these studies are not directly compatible on physical and language characteristics of stimuli (i.e. meaningful: [19, 13, 20]) versus meaningless stimuli [16–18], or on participants’ verbal abilities. More specifically, one major limitation of previous research is the inclusion of children with ASD with a much lower verbal IQ [11]: mean of 8.9 years with mean verbal age of 3.4 years; [21]: 9.4 years with verbal age of 4.9 years; 3: 10.5 yrs with verbal age of 5.8 years). Although passive listening paradigms were utilized, it is unknown whether differences in language abilities between TD children and children with ASD can account for some of atypicalities in speech versus non-speech brain responses found in previous studies. In line with this, the results of a recent MEG study suggested that atypical brain responses to both speech and non-speech in school-aged children with ASD were associated with poor language abilities in this population [22]. However, it remains unknown whether these atypical responses to speech and non-speech stimuli would be observed in children with ASD, who have the same verbal IQ as their TD peers. It is also worth noting that the majority of EEG/MEG studies in children with ASD were conducted in school-aged children (8 to 14 years) due to difficulties collecting neuroimaging data in younger children with ASD. To improve our understanding of the developmental trajectory of speech versus non-speech dysfunctions, due to the developmental nature of this disorder, it is important to investigate speech versus non-speech processing across different ages, especially in the younger groups.

Another major limitation to the existing ERP literature examining speech versus non-speech processing in ASD is a heavy reliance of the methodology previously employed on oddball paradigms. In particular, the MMN paradigm utilized in all of the ERP studies reviewed here relies on both habituation to a “standard” stimulus and dishabituation, reflected in a particular attentional orienting response (P3a) to a rarely presented “oddball” stimulus [8,11]. While the MMN paradigm is both well-established and powerful, there is both good reason and clear evidence indicating that the neural responses produced by this paradigm do not uniquely or directly reflect neural mechanisms associated with speech versus non-speech processing. Instead, MMN responses reflect a combination of perceptual and attentional network activity [23]. Furthermore, there is research evidence revealing atypicalities in both habituation [24] and attentional orienting neural networks [16] in individuals with ASD. Therefore, the almost exclusive reliance on the MMN in the autism speech and auditory processing literatures to date limits our current understanding of speech versus non-speech processing in this population.

In the current study, we employ a novel paired repetition paradigm designed to allow for a more direct and balanced assessment of the discrimination and detection of speech and non-speech stimuli in 4- to 6- year old children with ASD, when compared with a TD comparison group, individually matched on verbal abilities. A particular strength of this paradigm is that it does not recruit unrelated attentional orienting responses that may differ between the two participant groups. Specifically, we examine ERP responses to pairs of speech and non-speech sounds, including a speech sound followed by another speech sound, a non-speech sound followed by another non-speech sound, a speech sound followed by a non-speech sound, and a non-speech sound followed by a speech sound. The stimuli were phonetic sounds (/ba/, /da/, /ga/) and non-phonetic analogues for these speech sounds that were carefully matched to the speech sounds with regards to their physical characteristics. The aim of the study was to examine the discrimination of speech and non-speech from one another as perceptual categories of stimuli, through examining match and mismatch effects associated with these four types of stimulus pairings.

Based upon previous findings [11, 13, 22]; see also [25]for a review), we predicted between-group differences in the responses to speech and non-speech sounds; these would be reflected in ERP components associated with early cognitive processing of speech (e.g., 300 ms post-stimulus), associated with the recognition and classification of auditory stimuli [21, 25, 26]. Due to the nature of the paired repetition ERP paradigm employed here, we expected between group differences in speech processing to be reflected in responses to the Speech Mismatch versus Speech Match trials (Speech Mismatch effect), as well as in responses to Nonspeech Mismatch versus Nonspeech Match trials (Nonspeech mismatch effect). Since previous findings strongly suggest impairments/atypicalities in processing both speech and non-speech sounds in children with ASD [10, 14, 17, 21, 27, 28], we also predicted that control participants would exhibit mismatch effects both when non-speech sounds are followed by speech sounds, and when speech sounds are followed by non-speech sounds. However, we hypothesized that children with ASD would fail to exhibit match/mismatch effects in one or both of these conditions. Finally, we predicted atypical hemispheric lateralization of ERP responses to both speech and non-speech sound processing in children with ASD.

## Methods

### Participants

Fourteen intellectually able children with ASD (2 females, 12 males), and 11 TD children (2 females, 9 males), aged 4 to 6 years, as well as 3 TD younger children, aged 2 to 3 years (3 males), participated in the study. All 4- to 5- year old children with ASD who were included in the final sample had a verbal age of 40 months and above, based on the Mullen Scales of Early Learning (MSEL,[29]) assessment results. Both 6-year old children, included in the sample, completed the British Ability Scales assessment (BAS II, [30]) and had a verbal IQ of 70 and above. According to parent reports, all participants were English speakers and did not have significant exposure to any other language. Data from two additional participants initially recruited for the ASD group were excluded as their verbal age was observed to be below 40 months. Finally, data from one additional participant in the ASD group and two additional children initially recruited for the TD group were excluded due to prolonged exposure to a second language.

Twelve participants in the TD group were reported by their parents to be right handed, and two were left-handed; eleven participants with ASD were reported by their parents to be right handed, whereas three were left-handed. No child had a history of seizures or other medical or neurological disorder. All children had normal hearing and normal, or corrected to normal, vision. Eleven participants with ASD had an official diagnosis of an Autism Spectrum Disorder by a licensed clinical psychologist or medical doctor not associated with this research, and three other participants were in the process of obtaining a diagnosis. In all cases, a diagnosis of ASD was verified through the administration of Autism Diagnostic Observation Schedule–Generic (ADOS-G, [31]) in the laboratory by a formally trained research-reliable clinician. Based on the results of the ADOS assessment and expert clinical judgement, all children in the ASD group met clinical diagnostic criteria for an Autistic Spectrum Disorder. Data from one additional child initially recruited for the ASD group were excluded from the study as he did not meet cut-off criteria for ASD on the ADOS. In addition, the Social Communication Questionnaire (SCQ, [32]) was used as a second-level screening questionnaire for children with ASD and was completed by parents of all participants, in order to screen for social and communication difficulties in the TD control participants as well. No child in the control group received a score higher than 12.

To account for ERP differences potentially associated with verbal abilities, we utilized a comparison verbal age-matched control group (See **Table 1).** Children in the ASD and TD groups were matched individually, on one-to-one basis. In addition to verbal abilities, the ASD and TD groups did not differ in non-verbal abilities either. However, the two participant groups differed significantly in chronological age (CA) with a mean difference of 11 months (see Table 1). In order to control for the potential confounding effects of chronological age on the ERP effects, an analysis of covariance with CA as a covariate was computed separately for each ERP contrast. Similar analysis has been conducted before by Lepisto and colleagues (2005) in order to investigate the confounding effects of verbal age on the ERP effects [21]. The analyses revealed that CA did not have a significant linear relationship with any dependent variable (all F<3.2, ps>0.08). In other words, the ERP differences found between the groups were not affected by the differences in chronological age.

**Table 1:** Participants’ characteristics. Characteristics of children with ASD and typically developing (TD) participants individually matched on verbal mental age and the results of the group comparisons based on independent sample t-tests.

Characteristics	ASD group (n = 14)	TD group (n = 14)	Group comparison (p value)
Handedness	11 right, 3 left	12 right, 2 left	N/A
Gender	12 male, 2 female	12 male, 2 female	N/A
Chronological age in months (SD)	61 (8.8)	50 (11)	p = 0.02
MSEL and BAS verbal age in months (SD)	55 (10)	55 (13)	p = 0.82 (ASD vs TD VA)
MSEL and BAS non-verbal age in months (SD)	58 (10)	56 (13)	p = 0.57 (ASD vs TD VA)
ADOS communication sub-scale	3.9 (1.2)	N/A	N/A
ADOS social interaction subscale	6.5 (1.9)	N/A	N/A
ADOS total score	10.5 (2.9)	N/A	N/A

In accordance with the ethics protocol approved by the University of Birmingham, parents of all children who took part in the study reviewed and signed an approved consent form for their child to participate.

### Stimuli

The stimuli were created by utilizing the semi-synthetic speech generation method (SSG), and were also previously utilized in a study by Čeponiene and colleagues [33]. The SSG method allows to modify natural speech according to the aims of the particular study. It was shown that by utilizing natural glottal excitation generated by the fluctuation of vocal folds, the periodic structure of the synthesised waveform can achieve a realistic prosody and jitter [34].

Three consonant-vowel syllables, /ba/, /da/, and /ga/, spoken by a female English speaker, were recorded, digitized and used for computing the SSG in the current study. In particular, the glottal excitation waveform, the formant frequencies for the three consonants (/b/, /d/, /g/), as well as formant frequencies for the vowel /a/ were processed. Additionally, a 30 ms pre-voice bar which is normally present in the /ba/ syllables, was added to the /da/ and /ga/ stimuli in order to make the same gross structure of stimuli. Following pre-constant voice bar, the consonant burst lasted for 10 ms. The consonant-to-vowel 80 ms transition was then followed by an identical steady-state vowel /a/ which lasted for 60 ms. In total, the duration of the syllable and non-phonetic correlate stimuli were 180 ms. The non-phonetic correlates of the three speech syllables were created from five sinusoidal tones. Specifically, the frequencies and intensities of the tones were computed by the SSG and were chosen on the basis of the syllable format frequencies. The spectra of burst and burst-to-steady state format transitions, duration and intensities of the non-phonetic stimuli were kept equal to those of the corresponding natural speech stimuli [33]. As a result, synthesized stimuli were only different from corresponding speech stimuli in terms of their format transitions and plosives. The remaining acoustical features of the tones, including fundamental frequency, intonation and intensity duration were identical. Although speech and non-speech stimuli were very closely matched on physical characteristics, it has been shown that both adults and children perceive speech stimuli as speech, and non-speech stimuli as non-speech [33, 35].

### Procedure

In the current study, six different stimuli were used: three “speech” syllables (/ba/, /da/, and /ga/), and their three non-phonetic “non-speech” correlates. In total, there were four experimental conditions:Speech Match, Speech Mismatch, Non-Speech Match, and Non-Speech Mismatch. Each trial consisted of two sounds which were presented with an inter-stimulus interval of 50 ms. The presentation of the second stimulus was followed by a longer inter-trial interval which varied between 475, 550 and 625 ms. The trials were pseudo-randomized and were presented using E-Prime 2.0 software (Psychology Software Tools, Pittsburg, PA, USA). For each condition, an average of 430 trials was presented. The average number of trials did not differ between the two participant groups, or between experimental conditions (all F<2.3, ps>0.1, ASD group: Speech Match [204(73)], Speech Mismatch [204 (69)], NonSpeech Match [210 (67)], NonSpeech Mismatch [209 (71)]; TD group: Speech Match [206(66)], Speech Mismatch [191 (64)], NonSpeech Match [200 (63)], NonSpeech Mismatch [190 (61)].

Speech and non-speech stimuli were presented in a sound attenuated room via stereo speakers with a sound pressure level of 60 dB measured at the child’s head level. Children were seated in front of a computer monitor that presented a silent cartoon video of their choice, which was selected before the testing. The EEG recording and stimulus presentation lasted for approximately 30 minutes. Children were instructed to sit as still as possible while they were watching a silent video and the sounds were played in the background.

#### EEG recording

EEG was recorded continuously using a 128-channel Hydrocel Geodesic Sensor Net (Electrical Geodesics, Eugene, Oregon) with a sample rate of 500 Hz, referenced to a vertex electrode Cz. Electrode impedances were kept below 100 KOm. EEG data were processed offline using Netstation 4.4.1 software (Electrical Geodesics, Eugene, Oregon). The data were filtered (bandpass filter = 0.1–40 Hz) and segmented to epochs starting 100 ms before and continuing 800 ms after the presentation of the first auditory stimulus in the trial. The EEG data trials were further processed using an artefact detection tool that marked channels bad if the max-min threshold exceeded 100 mV and marked trials bad if they contained more than 12 bad channels. Individual electrodes were marked bad if they contained artefacts for more than 20% of recording. Following this automated procedure, remaining trials were also visually inspected by a trained observer and excluded from the analysis if they contained undetected bad channels, eye blinks and/or eye movements. Following artefact correction procedure, bad channels in the EEG data were replaced using a spherical spline interpolation algorithm [36]. The data were then averaged, re-referenced to an average reference for each participant and baseline corrected to a 100 ms pre-stimulus interval.

#### ERP components

Our primary hypotheses relate to perceptual and early cognitive processing mechanisms associated with the detection and categorization of speech versus non-speech (e.g., peaking between approximately 200 and 450 ms after stimulus onset). Because the speech and non-speech stimuli are closely physically matched with one another, we do not hypothesize or anticipate differences in auditory sensory components (e.g., peaking between 50 and 150 ms). Furthermore, because previous research indicates that auditory perceptual and early cognitive components are reflected in activity measured from the temporal and frontal-central electrodes, we focus our statistical analyses on temporal and frontal-central components peaking between approximately 200 and 450 ms (e.g., Frontal-central N250 and P350, Temporal P250 and N330). However, a comprehensive set of statistical analyses of both earlier and later ERP component activity is provided for the reader to have a more complete picture of early and late processing ERP components observed in the current study.

The ERP components recorded over the **frontal-central region** were as follows: a positive going component peaking at 140 ms (P150), a negative going component peaking at approximately 280 ms (N250), a positive going component peaking at 350 ms (P350) and a negative going Late Slow Wave (LSW) (N600). Over the **temporal region**, the ERP components observed included a negative going component peaking at 150 ms (N150), a positive going component peaking at approximately 280 ms (P250), a negative going component peaking at 330 ms (N330), and a positive going LSW (P600).

**Fig 1:**
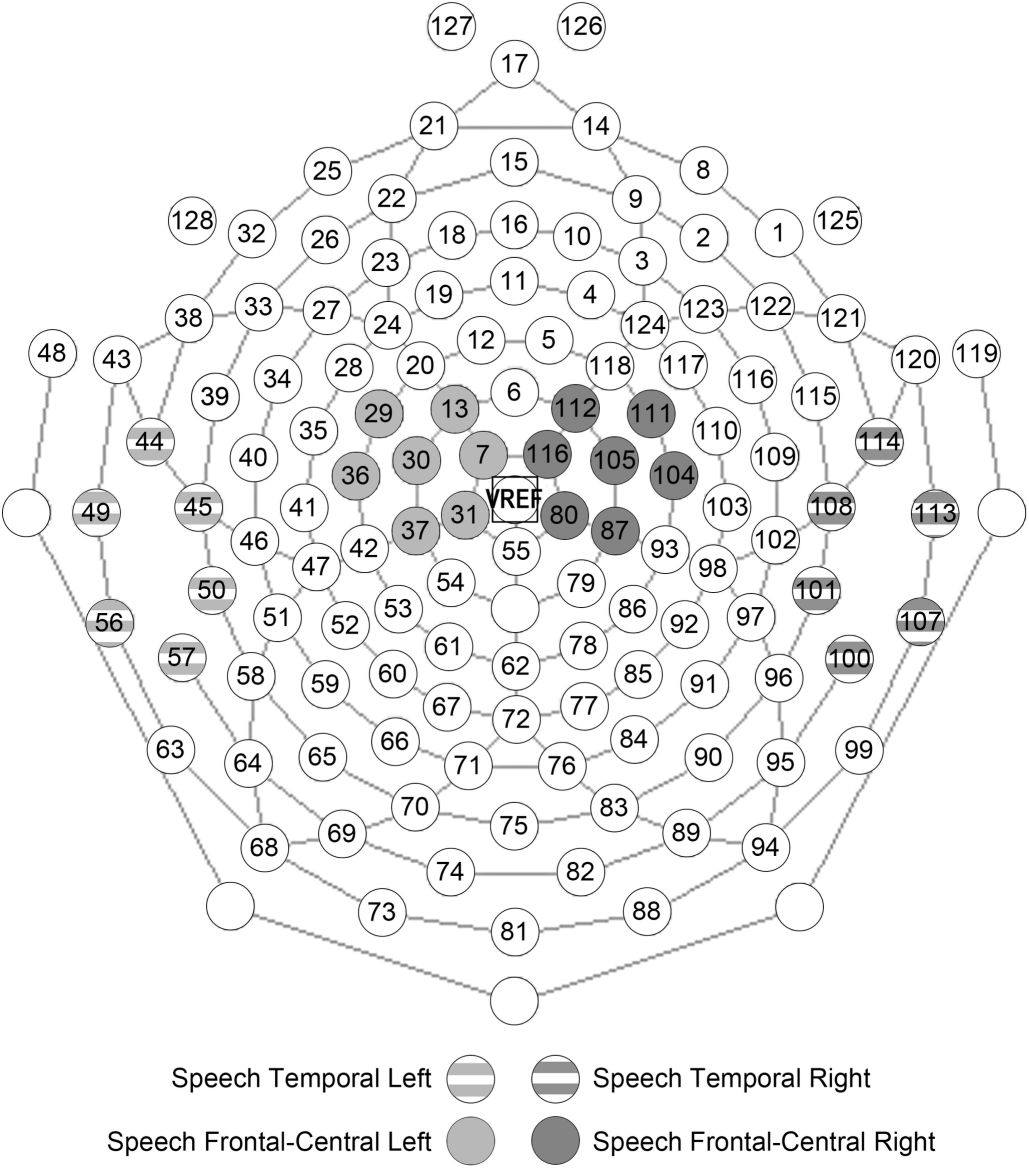
The electrode locations. The electrode locations in the frontal-central and temporal areas.

**Fig 2:**
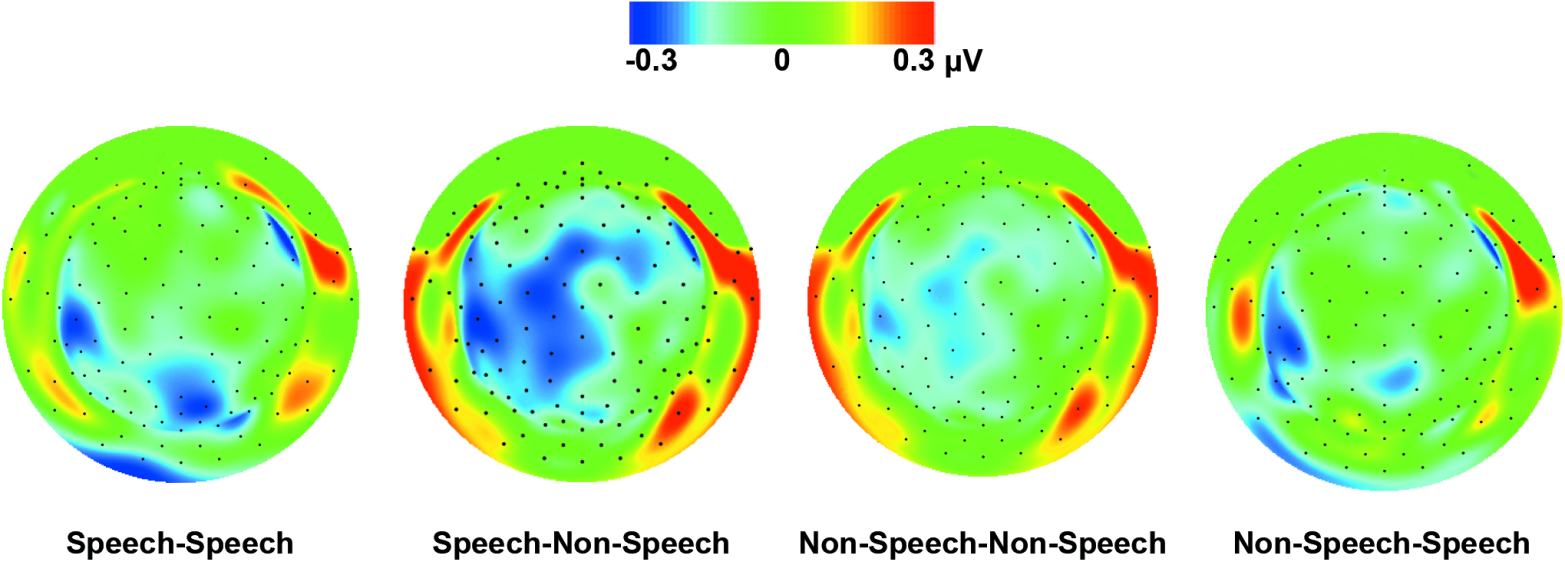
Topographical maps. Scalp potential distributions in the subtraction of topographical activity in the TD versus ASD groups in the selected time interval (300–400 ms) in the four experimental conditions.

Electrode locations included in the analysis were determined by visual inspection of individual data as well as grand average data of 14 participants with ASD and 14 control participants. Based upon our predictions and upon previous ERP findings of speech processing in ASD [8, 11, 12], 14 **frontal-central** (7 left hemisphere, 7 right hemisphere) and 12 **temporal** (6 left hemisphere, 6 right hemisphere) electrodes were identified for the analysis (see **Fig 1**). In addition, the location of temporal and frontal-central electrodes was verified based on the results of the difference plot, showing the scalp potential distributions in the time interval between 300 and 400 ms, in the TD group minus the ASD group (see **Fig 2)**. Peak amplitudes and latencies to peak amplitudes were analysed for all components except for the Late Slow Wave component, for which mean amplitudes were analysed. Time windows were selected for each component on the basis that the window encompassed the peak of the grand average for each condition, and also accurately measured the peak of the component for each condition for each individual participant. In cases, there were double-peaks for a component in individual ERP data, the time interval of the overall peak was considered for further analysis, in which the maximum and the minimum amplitudes of the double-peak were blindly determined by the program.

The following time windows were selected for each component in the **frontal-central** area: 110–190 ms (P150), 230–320 ms (N250), 300–420 ms (P350), 500–700 ms (N600). In addition, the following time windows were selected for each component in the **temporal area**: 100–200 ms (N150), 230–320 ms (P250), 300–430 ms (N330) and 500–700 ms (P600).

**Fig 3:**
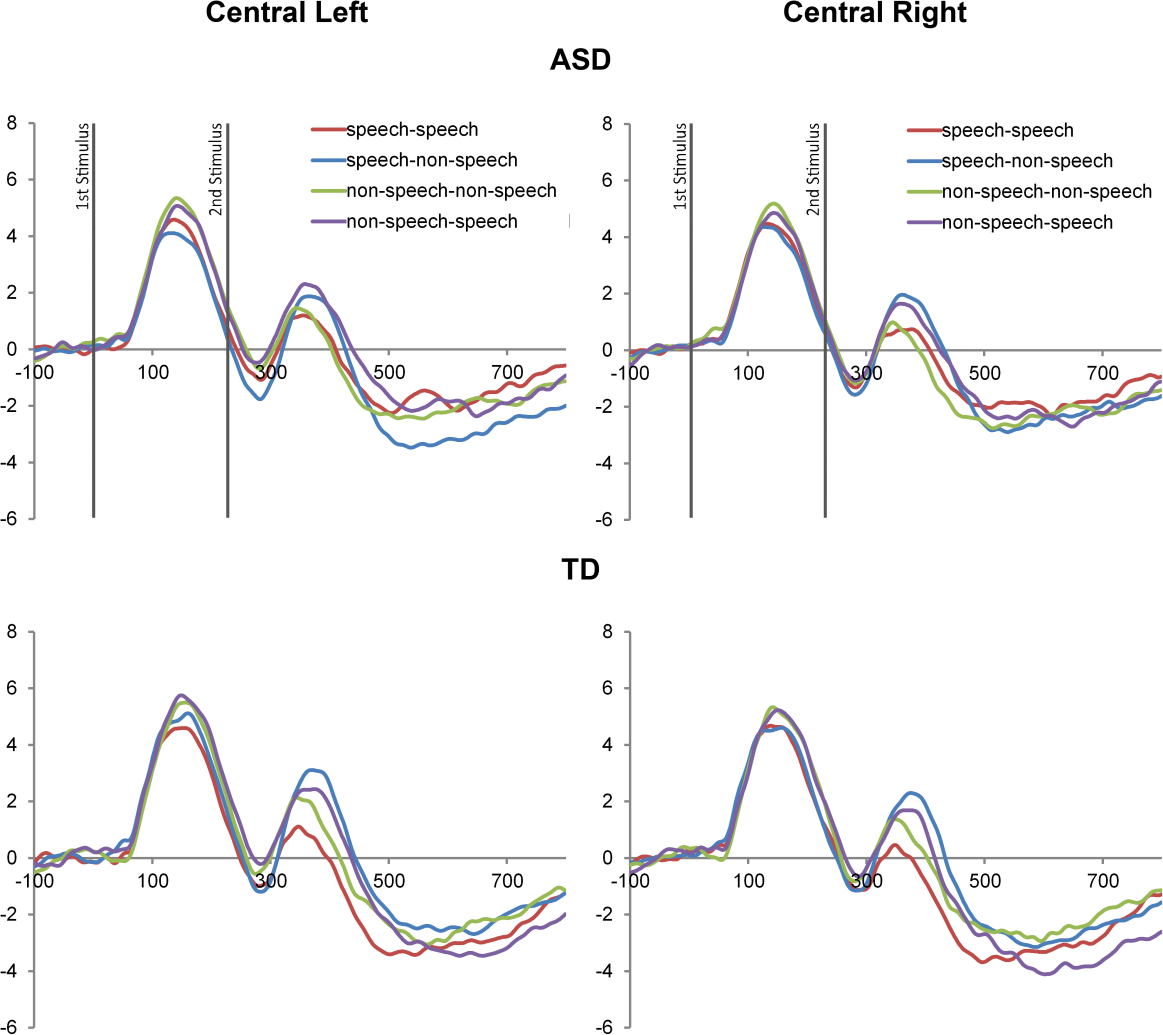
ERP waveforms in the frontal-central area. The figure represents the ERP waveforms recorded from the frontal-central electrodes in the left hemisphere (left side), and frontal-central electrodes in the right hemisphere (right side) in the ASD and the TD control groups.

**Fig 4:**
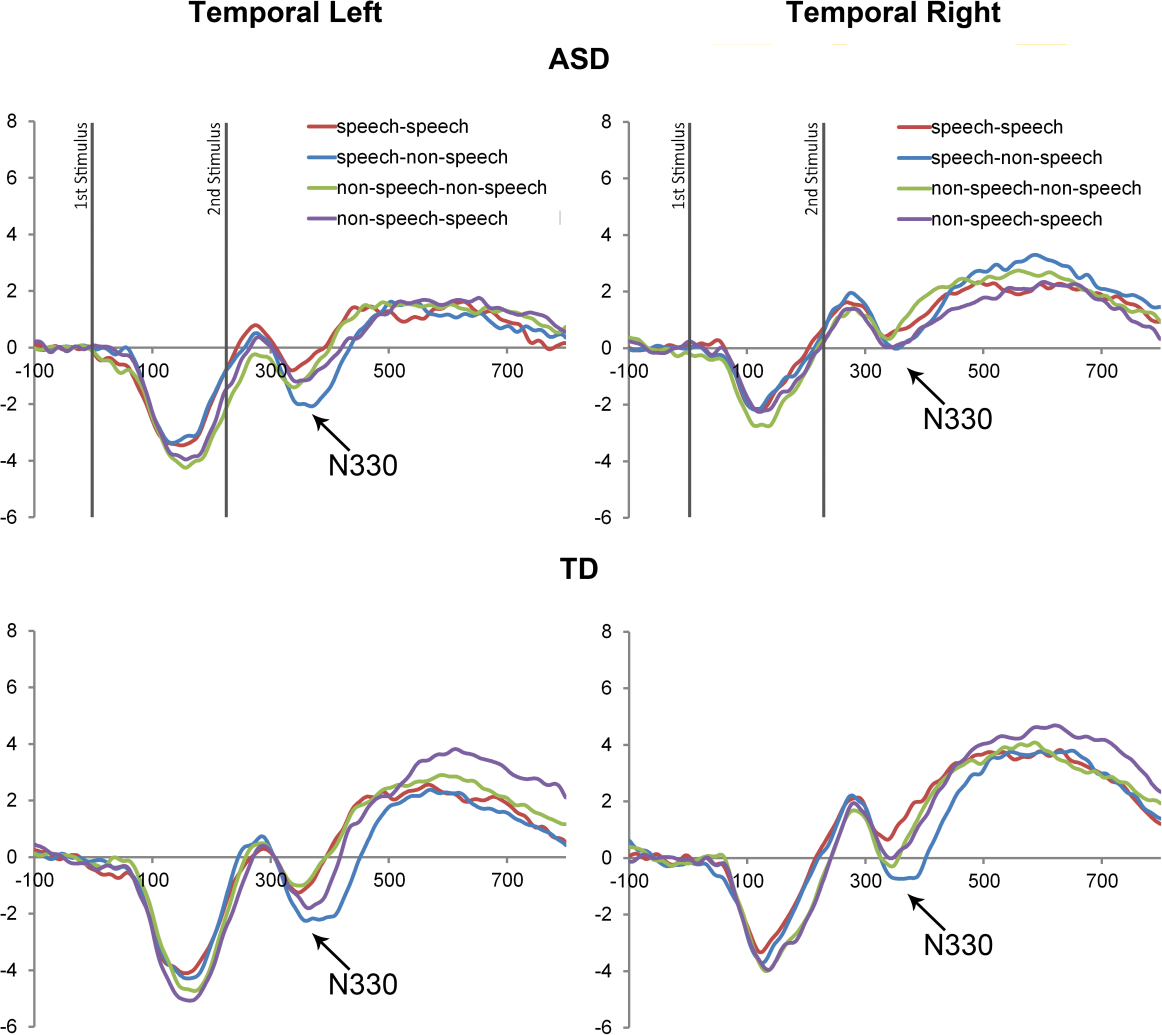
ERP waveforms in the temporal area. The figure represents the ERP waveforms recorded from the temporal left (left side) and temporal right (right side) electrodes in the ASD and the TD control groups.

Since analyses of covariance with CA as a covariate indicated no significant relationships between the CA and the dependent variables, the data were analysed with the repeated-measures analyses of variance (ANOVA). ANOVA with Stimulus (Speech vs Non-speech), Repetition (Match vs Mismatch), and Hemisphere (Left vs Right) as within-subject factors and Group (ASD vs TD) as a between-subject factor was performed on the peak (N150, P150, N250, P250, N330, P350) and mean (N600, P600) amplitudes and latencies of the aforementioned components over the frontal-central and temporal areas, separately. Post-hoc paired sample t-tests were carried out to explore further significant interactions that included factors of Stimulus, Repetition and Group, or Repetition and Group. Bonferroni corrections were employed for all post-hoc paired sample comparisons. The ERP waveforms for the ASD vs TD comparison group in **the frontal-central** and **temporal areas** are presented in **Fig 3 and Fig 4**.

## Results

### ERP effects–ASD vs TD group

#### Frontal-central components (Early perceptual (P150), Cognitive (N250, P350), Late Slow Wave (LSW) (N600))

A repeated measures ANOVA with within-subject factors of Stimulus (Speech vs Non-speech), Repetition (Match vs Mismatch), Hemisphere (Left vs Right) and a between-subject factor of Group (ASD vs TD) revealed a main effect of Stimulus for the latency (F(1,26) = 14, p = 0.001), and a main effect of Stimulus for the amplitude of the P150 component (F(1,26) = 4.4, p = 0.01). No other significant effects were observed for this component.

A main effect of Stimulus was found for the amplitude of the N250 component as well (F(1,26) = 16, p = 0.002). No further effects were observed for this component.

**Fig 5:**
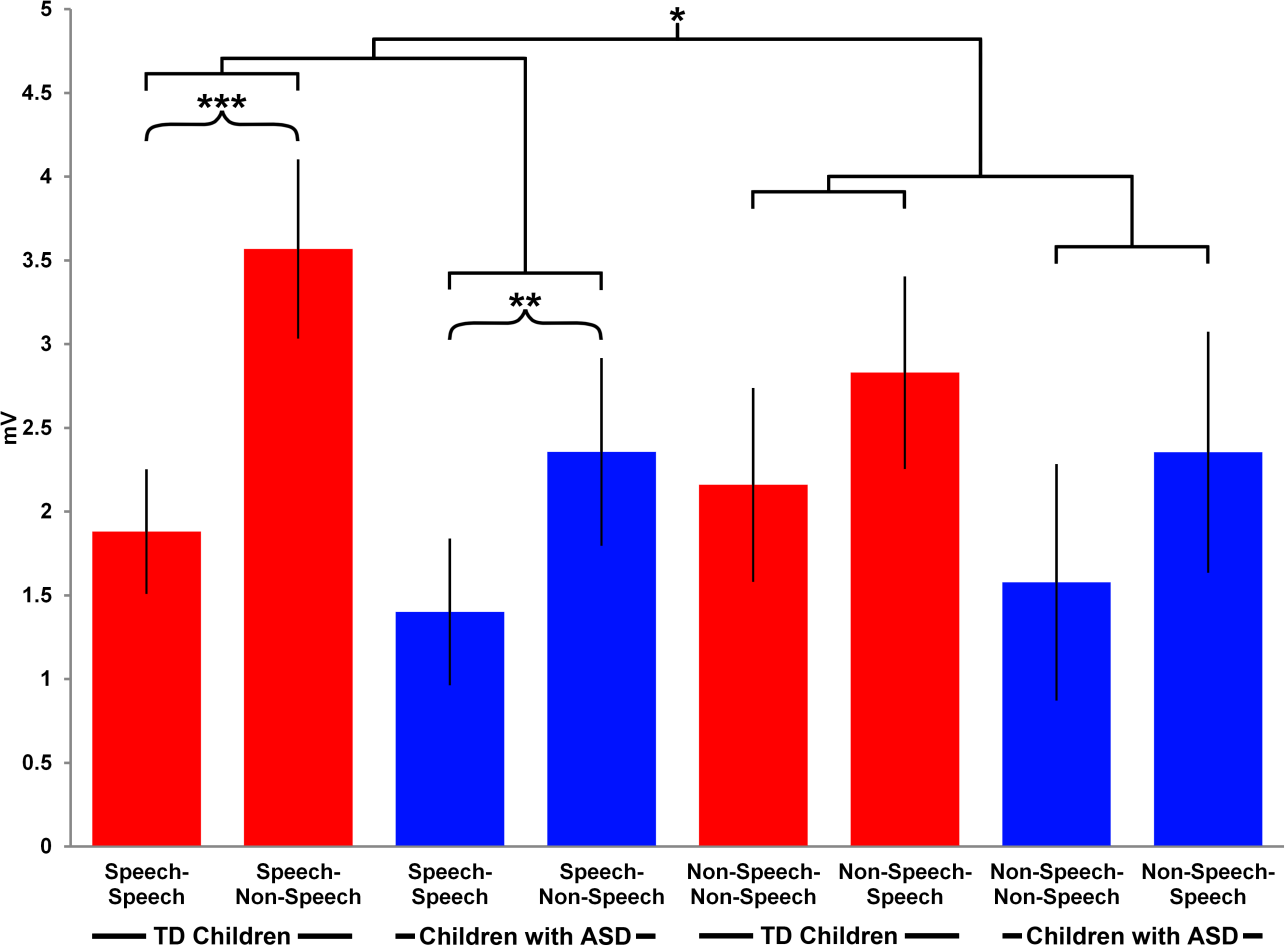
The P350 mismatch effect. The bar graph shows the mean ERP amplitudes for the P350 component in the fronto-central area in the four conditions the ASD and the TD groups. Asterisks indicate the results of post-hoc paired-sample t-tests: ***-p<0.001, **-p<0.01.

A repeated measures ANOVA with within-subject factors of Stimulus (Speech vs Non-speech), Repetition (Match vs Mismatch), and Hemisphere (Left vs Right), and a between-subject factor of Group (ASD vs TD) revealed a significant main effect of Repetition (F(1,26) = 26.7, p<0.0001) for the latency of the P350 component. Further analysis of the amplitude of the P350 component revealed a main effect of Repetition (F(1,26) = 36, p<0.0001), a significant interaction between Stimulus and Repetition (F(1,26) = 9.5, p = 0.01), as well as a significant interaction between Stimulus, Repetition and Group (F(1,26) = 5.2, p = 0.05). The post-hoc comparisons for the latter interaction showed a difference in processing matching versus mismatching speech sounds, with a higher amplitude to mismatching sounds in the condition, in which a speech stimulus was followed by a non-speech stimulus in both the TD (MD = -1.7, S.E. = 0.4, t = 5, df = 26, p<0.0001) and the ASD groups (MD = -1, S.E. = 0.4, t = 2.6, df = 26, p = 0.02) (see **Fig 5**).

**Fig 6:**
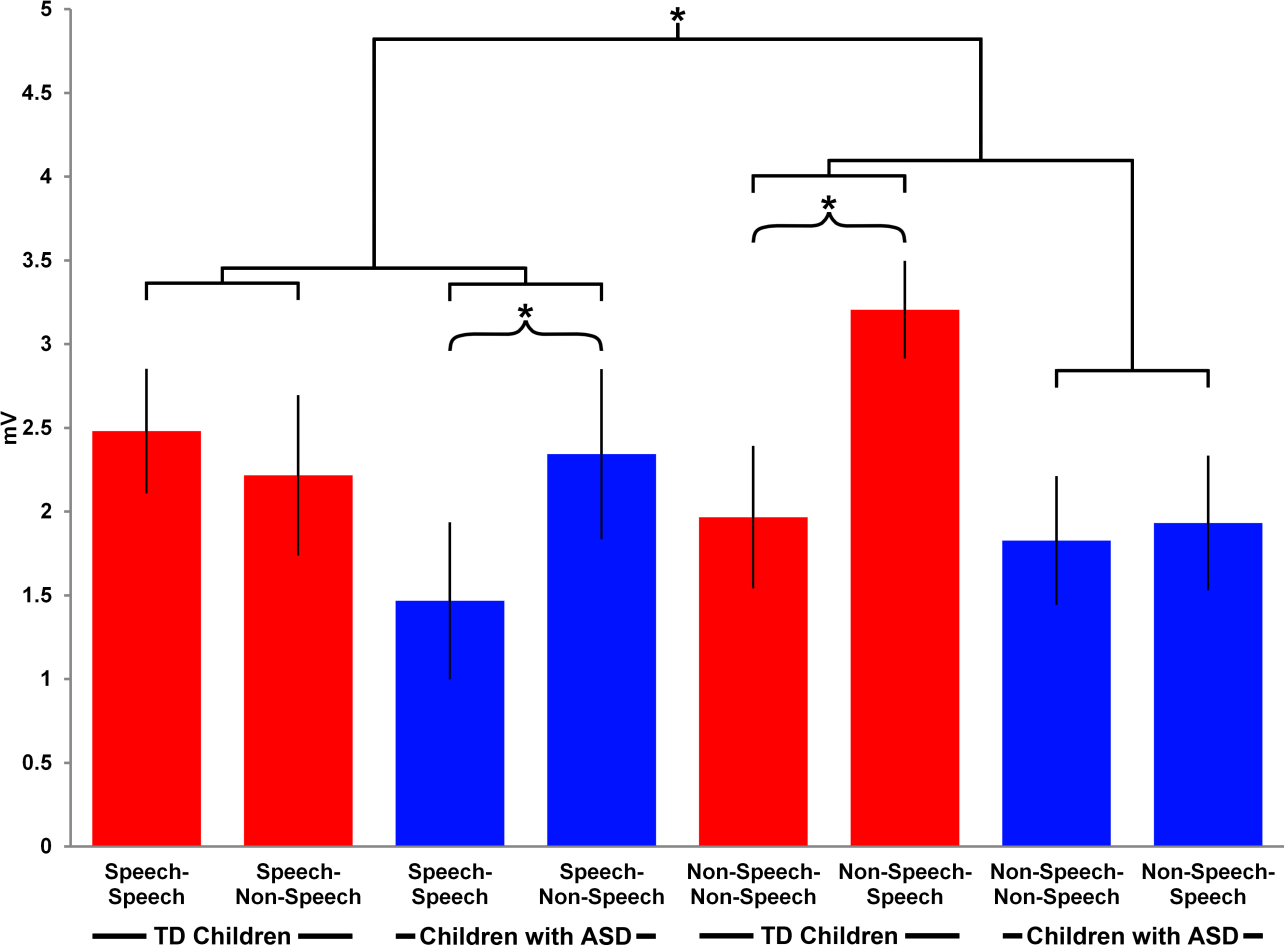
The N600 mismatch effect. The bar graph shows the mean ERP amplitudes for the N600 Late Slow Wave component in the central area in the four experimental conditions in the ASD and TD groups. The asterisks indicate the results of the post-hoc paired-sample t-tests: *—p<0.05.

There was also a main effect of Repetition (F(1,26) = 7, p = 0.01) and a significant interaction between Stimulus, Repetition and Group (F(1,26) = 6.5, p = 0.02) revealed for the amplitude of the N600 component. Follow-up t-tests for the latter interaction indicated a significant mismatch effect, i.e higher amplitudes to mismatch sounds, in the condition where a non-speech sound was followed by a speech sound in the TD group (MD = -1.2, S.E. = 0.4, t = 4, df = 26, p = 0.009) while the ASD group showed a mismatch effect in the condition, in which speech was followed by non-speech (MD = -0.9, S.E. = 0.3, t = 2.5, df = 26, p = 0.02) (see **Fig 6**).

#### Temporal components (Cognitive component (N330), Late Slow Wave component (LSW–P600)

**Fig 7:**
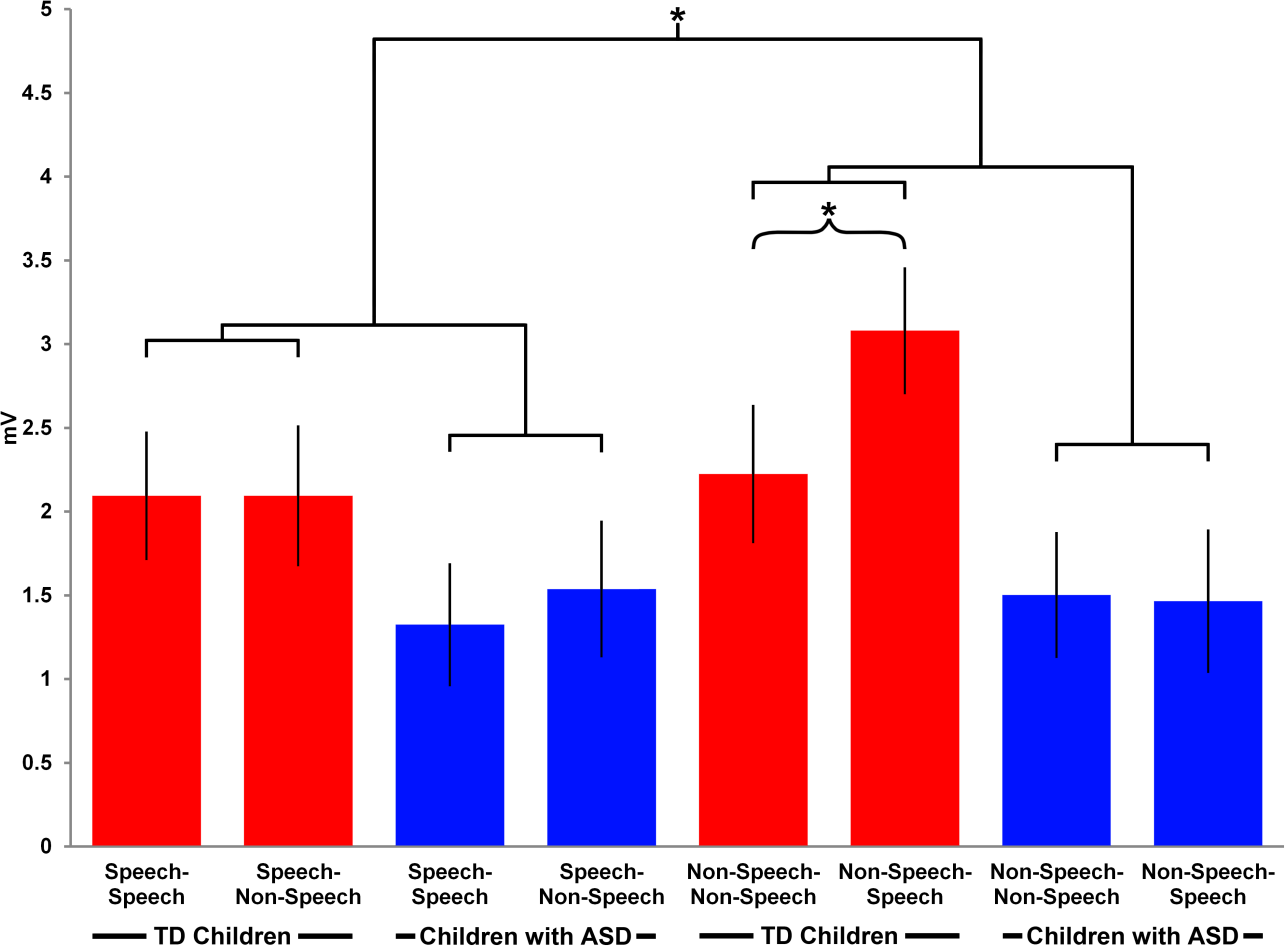
The P600 mismatch effect. The bar graph shows the mean ERP amplitudes for the P600 Late Slow Wave component in the temporal area in the four experimental conditions in the ASD and TD groups. The asterisks indicate the results of the post-hoc paired-sample t-tests:*—p<0.05.

A repeated measures ANOVA carried out on the amplitude of the N330 component revealed a main effect of Repetition (F(1,26) = 21.3, p<<0.0001), a significant interaction between Stimulus and Repetition (F(1,26) = 8.8, p = 0.006) and a significant interaction between Stimulus, Repetition, Hemisphere, and Group (F(1,26) = 5, p = 0.03). The post-hoc paired sample t-tests showed between-group lateralization differences for a mismatch effect, in the condition where a speech stimulus was followed by a non-speech stimulus. In particular, for the N330 component, the speech mismatch effect was present in both hemispheres in the TD group (MD = 1.3, S.E. = 0.3, t = 4.2, df = 26, p = 0.001; MD = 1.6, S.E. = 0.5, t = 4.1, df = 26, p = 0.002), while the ASD group exhibited the same effect in the left hemisphere only (MD = 1.2, S.E. = 0.3, t = 3.9, df = 26,p = 0.001) (see **Fig 4**). For the latency of the N330 component, there was a significant main effect of Stimulus (F(1,26) = 6.5, p = 0.04) and a main effect of Repetition (F(1,26) = 66.5, p<0.0001). For the amplitude of the P600 component, a main effect of Stimulus (F(1,26) = 4, p = 0.05), a significant interaction between Stimulus, Repetition and Group (F(1,26) = 6.1, p = 0.03) as well as the between-subject factor of Group were revealed (p = 0.03). The significant interaction between Stimulus, Repetition and Group indicated a mismatch effect in the condition where non-speech was followed by speech in the TD group (MD = 1, S.E. = 0.3, t = 5.1, df = 26, p = 0.002), while the ASD group did not exhibit any differences (p>0.1) (see Fig 7). Finally, a between subject factor of Group indicated reduced P600 activity in the ASD group compared to the TD group (p = 0.03).

## Discussion

In the current study, we investigated neural responses to speech and non-speech sounds in children with ASD and TD children, individually matched on verbal age, using a paired repetition ERP paradigm. The goal of this study was to further our understanding of the neural mechanisms underlying the detection and processing of speech in this population. To our knowledge, this is the first ERP study to investigate the detection and discrimination of speech from non-speech in 4- to 6- year old children with ASD without the use of oddball stimuli and associated attentional orienting responses. The current findings add novel information to the existing literature and research findings observed in previous studies, conducted in school-aged children and using MMN or variations of MMN paradigms [11,12, 21,25, 26, 28]. In addition, this is the first study to control for differences in verbal abilities between children with ASD and the TD group. This means that the current findings indicate differences in brain functioning between the groups that may be associated with the atypical development of communication and social skills observed in ASD, rather than differences in language ability between the groups. More specifically, the current study adds to existing literature exploring the relationship between speech versus non-speech processing dysfunctions and language delays [22, 26, 37], or social versus non-social processing abnormalities revealed in this population [4,5,8,9,15, 38,39]. It is also worth noting that the results of an additional covariate analysis showed that chronological age did not affect differences in the ERP components.

The current data revealed that speech mismatch effects, indexed by higher ERP amplitudes to mismatch sounds in the condition where speech was followed by non-speech in the temporal N330 and central P350 components, were present in both the ASD and TD groups. However, we also observed significant between-group differences in the lateralization of the speech mismatch effect in the N330 component. Specifically, the N330 mismatch effect was present over both the right and left temporal electrodes for the TD group, whereas the ASD group exhibited the same effect over the left temporal electrodes only. It is worth noting that both of these components exhibited a mismatch effect in both the TD and ASD groups, and only in the conditions where a speech stimulus was followed by a non-speech stimulus. This finding of a largely intact speech versus non-speech mismatch effect in the ASD group allows us to conclude that children with ASD spontaneously discriminate non-speech sounds if preceded by speech. This is consistent with previous findings indicating that children with ASD can attend to and detect the acoustic changes in non-speech sounds, which exhibits the same or enhanced ERP responses, relative to control participants [14, 21, 26].

In additional analyses carried out on early sensory components, no statistical group differences were observed, most likely due to the fact that our speech and non-speech stimuli were closely physically matched, and therefore did not trigger sensory-perceptual component response differences. By closely matching speech and non-speech stimuli, we significantly reduced the possibility that uncontrolled differences in the physical characteristics of the stimuli could have impacted upon group differences in speech versus non-speech detection and discrimination in the current study.

Furthermore, additional analyses of late cognitive processing revealed significant between-group differences in the late cognitive temporal P600 and central N600 components. Specifically, the TD participant group exhibited mismatch effects during trials where non-speech stimuli were followed by speech stimuli. These mismatch effects were absent in the ASD group for both components. However, the ASD group exhibited a reverse mismatch effect, whereby their N600 component responses indexed a mismatch response during trials where speech stimuli were followed by non-speech stimuli. Taken together, these findings suggest that in a context where a non-speech stimulus is presented first, the ASD group exhibits reduced cognitive evaluation of the fact that a subsequent speech sound is different from the preceding non-speech sound. It is also worth mentioning that, since we have not predicted these LSW effects (N600, P600), which have not been found in previous studies of speech processing in ASD [8,11,12, 21, 26, 37], our interpretation of the LSW effects should be taken into account with caution, as more studies need to be conducted in order to investigate the nature of the N600 and the P600 mismatch effects.

Overall, the current findings in younger children are consistent with previous research in older school-aged children suggesting that between-group differences in speech versus non-speech processing are associated with late cognitive ERP component activity (after 300 ms) recorded from temporal and frontal-central electrodes [8,14, 22, 26]. In addition, the current findings suggest that the detection and discrimination of speech and non-speech stimuli from one another in children with ASD are associated with atypical hemispheric activation, which may reflect failure to recruit particular neural mechanisms for this task.

As mentioned above, the ERP differences representing speech/non-speech mismatch effects were absent for early sensory-perceptual ERP components (N150, P150), as well as for early cognitive components (N250, P250). Together, the timings of the revealed mismatch effects suggest that these effects were driven by cognitive processing of stimuli (N330, P350), followed by a later cognitive evaluation of speech and non-speech stimuli (N600, P600), recorded from temporal and frontal-central electrodes. Thus one possible interpretation of the presence of the speech mismatch effects at the N330, the P350 and the N600 components, and the absence of the non-speech mismatch effects at the N600 and the P600 components in children with autism is that they are able to identify and process a *non-speech* sound preceded by a speech sound at approximately 330 ms to 350 ms and 600 ms, but fail to distinguish a *speech* sound from a preceding non-speech sound during the late cognitive evaluation stage. These findings may reflect how young children with ASD categorize and consider speech and non-speech to be different from one another. Importantly, they further support the notion that speech versus non-speech processing is not universally impaired in ASD, and the nature of the task and the order of speech and non-speech stimuli matters for the successful differentiation of stimuli [12, 25]. The presence of speech mismatch effects in the ASD group (N330, P350 ms) also supports the view that individuals with ASD might not have an impaired speech processing mechanism as such, but use different neurophysiological mechanisms and strategies for processing speech and non-speech sounds [14, 25]. This suggestion also fits with a previous hypothesis that children have a specific auditory processing impairment, according to which they fail to recognise the greater meaning of speech over the non-speech or synthesized speech, most likely by inhibiting the natural attention to speech stimuli [8, 22, 40].

Along with the diminished cognitive evaluation of speech sounds in the present study, children with ASD also exhibited atypical lateralization revealed for the temporal N330 component. More specifically, the TD group exhibited bilateral mismatch effects in the condition where speech was followed by a non-speech sound, while the ASD group demonstrated a *left* lateralized mismatch effect in the same condition. This finding is consistent with the previous EEG and fMRI research on this population. In particular, Stroganova and colleagues uncovered evidence for reduced pre-attentive processing of simple tones in the right-hemisphere in children with ASD [16]. Furthermore, two neuroimaging studies have reported increased activation of left temporal cortex in children with ASD during semantic processing [41] and song processing [42] tasks. On the other hand, Redcay and Courchesne (2008) observed increased right hemisphere activation during speech processing in toddlers with ASD. In contrast to the speech stimuli utilized in that study [19, 43], however, the speech and non-speech stimuli utilized in the current study were both meaningless and very closely matched on their physical characteristics.

With regards to the reliance of TD participants on both hemispheres, and ASD participants solely on the left hemisphere, for the detection and discrimination of speech, it seems possible, based upon previous research, that right temporal activity observed in the TD group in the current study may reflect processing of non-phonetic speech characteristics, such as prosody, intonation, and other social-emotional aspects of speech. This notion is consistent with a recent model suggesting two distinct functions of left and right hemisphere for speech processing [44, 45]. Consistent with this hypothesis, Boddaert and colleagues found that listening to complex synthetic speech-like stimuli can elicit abnormal cortical processing in children with autism in a similar fashion as to speech stimuli [46]. Taken together, the evidence to date suggests atypical processing of prosody and emotional prosodic cues in speech [13, 27] and deficient processing of vocal sounds, both speech and non-speech, in children with ASD [28]. By demonstrating atypical lateralization of the speech mismatch effect, the current results support the conclusions from a recent ERP study suggesting that atypicalities in responses to speech versus non-speech sounds might be related to the delayed maturation of vocal sound processing, either speech or non-speech [28], most likely due to the social nature of the stimuli. Therefore, the lack of right temporal activity in the ASD group in the current study may be associated with diminished processing of socio-emotional aspects of speech, such as prosody and intonation, which commonly occurs in the right hemisphere in typical individuals.

### Limitations

Due to the nature of the paired-repetition paradigm, in which the two sounds were presented only 50 ms apart, there is some overlap between speech and non-speech sound processing in each condition. Hence, the waveform activity shown in **Fig 3** and **Fig 4** represent the ERP responses to both sounds after the presentation of the second stimulus. Despite the overlap affects the waveform figures, there is no risk in contaminating our statistical results. Since the first sounds in both match and mismatch conditions are the same, the overlap has the same impact on the ERP waveform in these two conditions. Therefore, this effect will be cancelled out when we consider the difference between match and mismatch conditions in each group.

Another potential limitation of the current study is the concurrent presentation of visual (silent video) and auditory stimuli in the experimental paradigm. It has been suggested that the concurrent presentation of visual stimuli during the auditory ERP recording helps to reduce artefacts, created by body and eye movements [47]. It is worth noting, however, that visual stimuli in cartoon videos did not contain associations with presented auditory speech and non-speech sounds and were not synchronised with them. Despite this precaution, it was not possible to avoid the fact that the silent cartoon videos included scenes of social interaction and facial/mouth motion. Video presentation however is now a common practice instead of puppet shows, which were traditionally used in the studies of auditory processing in young children and are believed to be less experimentally controlled than videos [48].

Finally, since the paired repetition paradigm included passive listening, we cannot be certain about whether children with ASD perceived speech sounds as speech, and non-speech sounds as non-speech. As mentioned in the Methods section, results from previous studies utilizing the same stimuli suggested that both adults and children [33, 35] can correctly discriminate speech from non-speech sounds. Although the stimuli employed in the present study are similar to those used in the Ceponiene et al. [33, 35] experimental paradigms, the discrimination of the two types of stimuli was not tested in our child participant sample through the use of a behavioural measure.

### Conclusions

In summary, the current study utilized a paired repetition paradigm that is unique in that it does not rely on oddball stimulus presentation which elicits attentional orienting responses, and therefore confounds the measurement of speech and non-speech processing mechanisms. Results provide evidence that while children with ASD initially detect and discriminate the difference between speech and non-speech stimuli at the same stage (N330, P350) as TD control participants, they rely solely on the left hemisphere when doing so. The failure to recruit right temporal cortex for this purpose likely reflects significantly reduced reliance on social-emotional cues for speech which are typically reflected in activity recorded from right temporal electrodes. These findings suggest that even when children with ASD distinguish speech and non-speech stimuli from one another, they do not consider or evaluate this distinction in the same manner as TD children do.

Together, the current findings provide novel information to implicate a role for impaired or atypical recruitment of the right hemisphere for speech versus non-speech detection and discrimination, and further suggest particular relationships between reduced reliance on social-emotional processing mechanisms and failure to evaluate the distinction between speech and non-speech stimuli at later cognitive stages of processing. These findings help to shed light on the neural mechanisms that may underlie the well-documented failure to behaviorally orient to speech stimuli in this population, as well as potential pathways from impaired social-emotional functioning to impaired speech versus non-speech detection and discrimination. Future research should be conducted to examine potential relationships between behavioural orienting responses for speech and non-speech and the neural mechanisms uncovered in the current study. Furthermore, given the high applicability and adaptability of this passive auditory event-related potentials paradigm for use with infants and for individuals who are nonverbal and minimally verbal, future research applying this procedure to multiple populations has a great potential to provide insights into both the developmental time-course and the impact of these mechanisms on speech processing and language-learning in individuals with autism.
